# Heat Shock Protein Family A Member 1A Attenuates Apoptosis and Oxidative Stress via ERK/JNK Pathway in Hyperplastic Prostate

**DOI:** 10.1002/mco2.70129

**Published:** 2025-03-10

**Authors:** Huan Liu, Yongying Zhou, Zhen Wang, Daoquan Liu, Yan Li, Huan Lai, Jizhang Qiu, Shidong Shan, Feng Guo, Ping Chen, Yuming Guo, Guang Zeng, Michael E. DiSanto, Xinhua Zhang

**Affiliations:** ^1^ Department of Urology Zhongnan Hospital of Wuhan University Wuhan China; ^2^ Department of Urology Ningbo Medical Center LiHuiLi Hospital of Ningbo University Ningbo China; ^3^ Department of Thoracic Surgery Zhongnan Hospital of Wuhan University Wuhan China; ^4^ Department of Surgery and Biomedical Sciences Cooper Medical School of Rowan University Camden New Jersey USA

**Keywords:** apoptosis, benign prostatic hyperplasia, heat shock protein family A member 1A, oxidative stress, prostate‐specific antigen

## Abstract

Benign prostatic hyperplasia (BPH) is a prevalent disorder in aging males. It is investigated whether heat shock protein family A member 1A (HSPA1A), a cytoprotective chaperone induced under stress, has been implicated in the development of BPH. RNA‐sequencing and single‐cell sequencing analyses revealed significant upregulation of HSPA1A in BPH compared to controls. In vitro experiments elucidated that HSPA1A was localized in prostatic epithelium and stroma, with upregulated expression in BPH tissues. Moreover, HSPA1A silencing augmented apoptosis and reactive oxygen species (ROS) accumulation, inhibiting proliferation via ERK/JNK activation, while overexpression reversed these effects in prostatic BPH‐1 and WPMY‐1 cells. Additionally, ERK1/2 suppression with U0126 rescued the effects of HSPA1A silencing. In vivo, testosterone‐induced BPH (T‐BPH) rat models treated with the HSPA1A antagonist KNK437 exhibited prostatic atrophy and molecular changes consistent with reduced HSPA1A activity. Finally, we conducted a tissue microarray (TMA) analysis of 139 BPH specimens from Zhongnan Hospital of Wuhan University, which revealed a positive correlation between HSPA1A expression and clinical parameters, including prostate volume (PV), tPSA, fPSA, and IPSS. In conclusion, our findings suggested that HSPA1A attenuated apoptosis and oxidative stress through the ERK/JNK signaling pathway, contributing to BPH pathogenesis.

## Graphical abstract

Upregulation of HSPA1A increased the level of Bcl‐2 and antioxidants by suppressing the phosphorylation of the ERK/JNK cascade, thereby enhancing cell proliferation. However, the reduction of HSPA1A associated with misfolding of peptides promoted the transcription of BAX and Caspase‐3 via activating ERK/JNK, leading to cell apoptosis and ROS accumulation. Consequently, the imbalance between cell proliferation and cell apoptosis and ROS induced by the HSPA1A–ERK/JNK cascade contributed to the development of BPH.

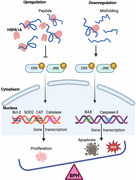

## 1 Introduction

Benign prostatic hyperplasia (BPH) is a prevalent nonmalignant disorder that affects aging men. In elderly males over 80 years old, the morbidity rises to as high as 80% worldwide [1]. Lower urinary tract symptoms (LUTS) and bladder outlet obstruction (BOO), common consequences of BPH, significantly impair the well‐being of patients [2]. Existing evidence suggests that uncontrolled proliferation of epithelial and stromal cells or dysregulated programmed cell death in the prostatic transitional zone may contribute to the development of BPH/LUTS [3]. Various hypotheses, including imbalances in testosterone and estrogen levels [4], stromal–epithelial interactions [5], and embryonic reawakening [6], have been proposed to elucidate the emergence and progression of BPH. In addition, the androgen receptor (AR) cascade plays a vital role in suppressing prostatic inflammation, while disruption of which in luminal cells could induce immune cell infiltration and cell proliferation, thereby contributing to the development of BPH [7]. However, a clear consensus on the in‐depth mechanisms underlying the etiopathogenesis of BPH remains elusive.

With the vigorous expansion of bioinformatics analysis, numerous studies have shed light on the pathophysiology mechanisms related to BPH [8]. Notably, differentially expressed genes (DEGs) screened out via high‐throughput RNA sequencing (RNA‐seq) have garnered significant attention for their potential roles in elucidating cause‐and‐effect relationships, identifying biological markers, or developing therapeutic approaches for various diseases, including BPH. Liu et al. conducted an in‐depth molecular analysis of BPH and discovered two distinct molecular subgroups. This investigation encompassed genomic, transcriptomic, and epigenetic profiling [9]. Similarly, a more recent study explored potential therapeutic targets for BPH by utilizing single‐cell RNA sequencing (scRNA‐seq) data [10]. Previously, our group conducted an analysis of mRNA expression profiling based on three normal and five hyperplastic human prostate samples and identified 207 DEGs, including upregulated genes like *BMP5, NELL2, CXCL13, and NRK*, and downregulated genes like *GPX3* [11, 12, 13, 14, 15]. While most of them have been proposed for further investigation, heat shock protein family A member A (HSPA1A), one of the most highly expressed DEGs, remains obscure in the development of BPH.

The HSP70/HSPA family is the largest subfamily of heat shock proteins, comprising 7 and 13 homologs in mice and humans, respectively [16]. HSPA1A, also known as HSP70‐1 or HSP72, belongs to the HSP70 family and functions as the cytoplasmic stress‐inducible form [17]. HSPA1A consists of a nucleotide‐binding domain (NBD) and a substrate‐binding domain (SBD) connected by a linker region. This linker couples the exchange of ADP for ATP with the release of the bound protein substrate [18]. Under physiological conditions, HSPA1A exhibits low expression levels. However, its expression is highly induced under environmental stress conditions, such as anoxia, ischemia, and heat shock, which can result in inappropriate folding of cytosolic proteins [19]. Upregulation of HSPA1A has been observed in a variety of diseases, including Type 2 diabetes, Alzheimer's disease, various cancers, chronic obstructive pulmonary disease, prostatitis, or urinary tract infection [20, 21, 22, 23]. A wide range of studies have identified the cytoprotective effects of HSPA1A in regulating apoptosis and cell growth. Upregulation of HSPA1A has been shown to prevent cell death by inhibiting apoptotic or necroptotic pathways, as well as stabilizing inhibitors of apoptosis proteins (IAPs) [24]. In vivo studies have demonstrated that HSPA1A inhibits H_2_O_2_‐induced apoptosis, leading to the recovery of neurological function after spinal cord injury [25]. Furthermore, this molecule plays a crucial role in orchestrating cellular signaling and protein degradation, thereby enhancing cell viability in various cancers, including liver, prostate, and breast cancer [26, 27, 28]. In addition to its role in apoptosis and cell growth, HSPA1A also displays a correlation with oxidative stress (OS). Overexpressed HSPA1A in transgenic mice has been associated with reduced liver injury and lower OS levels [29]. In cerebral microvascular endothelial cells, the knockdown of HSPA1A has been shown to increase the production of reactive oxygen species (ROS) by counteracting the effects of fibroblast growth factor 21 (FGF21) under hypoxic conditions [30].

Despite emerging evidence suggesting the pro‐survival role of HSPA1A, the interplay between HSPA1A and BPH has not been elucidated in the literature thus far. In this study, we revealed an elevated expression level of HSPA1A in human BPH tissues, corroborated by integrative bioinformatics analysis. Furthermore, we demonstrated that HSPA1A could suppress cell apoptosis and ROS accumulation in BPH cell models and T‐BPH rat models through the ERK/JNK pathway. Moreover, our findings identified a positive correlation between HSPA1A expression levels and clinical indicators of BPH. Taking together, we hypothesized that HSPA1A could suppress apoptosis and OS via the ERK/JNK pathway in the progression of BPH.

## 2 Results

### 2.1 RNA‐Seq and Single Cell Sequencing Analysis Between BPH and Control Groups

**FIGURE 1:**
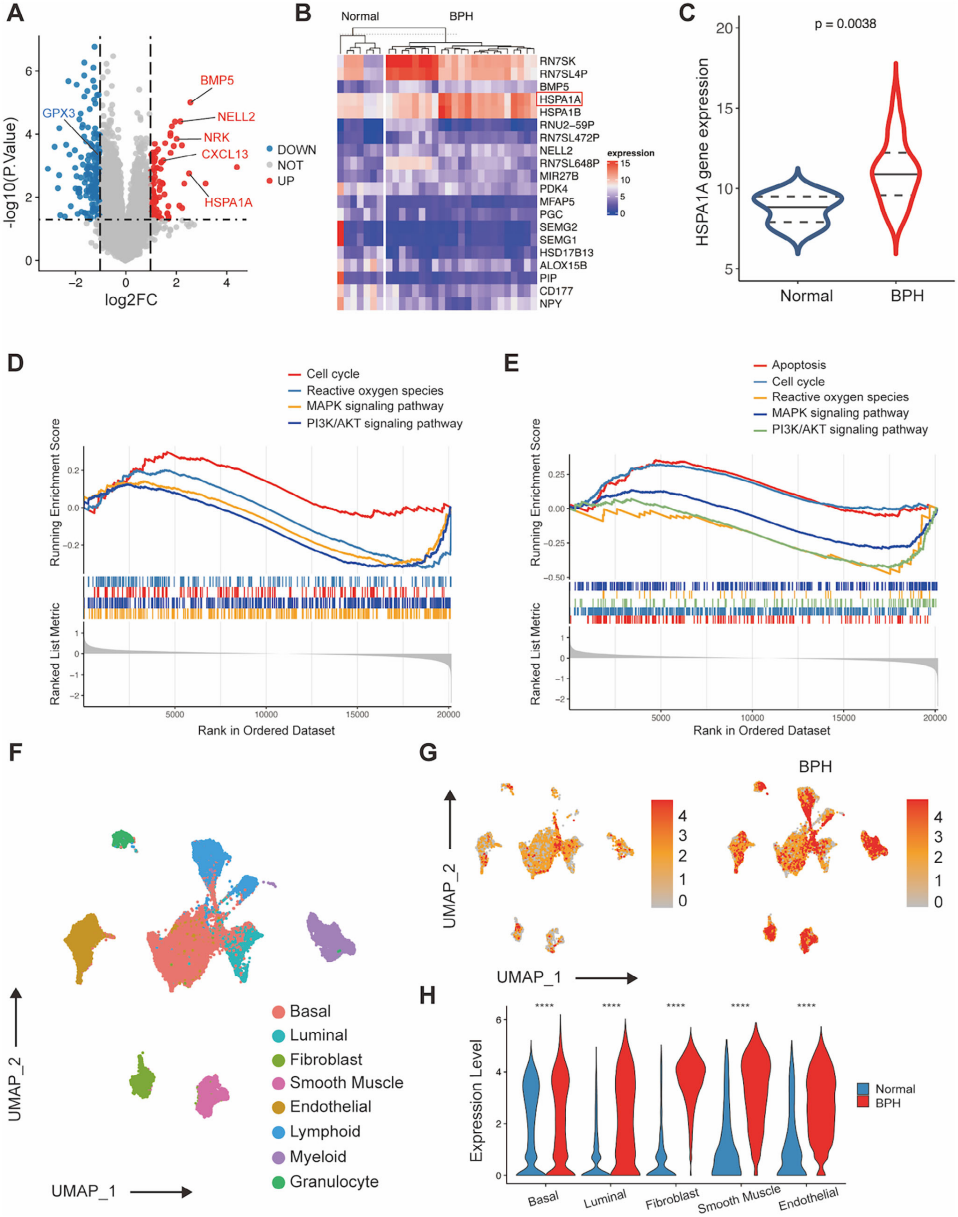
Identification of HSPA1A as a potential target in BPH. (A) Volcano plot of DEGs in an integrated dataset of GSE119195 and GSE132714. (B) Heatmap of top DEGs in the integrated dataset of GSE119195 and GSE132714. (C) The expression level of HSPA1A in human normal prostate tissues versus BPH tissues in the integrated dataset of GSE119195 and GSE132714. (D, E) GSEA analysis of the integrated dataset of GSE119195 and GSE132714. (F) Cell types of single‐cell analysis. (G) Feature plot of HSPA1A expression in normal and BPH samples of single‐cell data. (H) Expression of HSPA1A in basal cells, luminal cells, fibroblasts, smooth muscle, and endothelial cells.

Two datasets, GSE119195 and GSE132714, obtained from the Gene Expression Omnibus (GEO) database, were integrated and then subjected to RNA‐seq analysis. Analysis of this integrated dataset revealed 260 DEGs, including 91 upregulated genes such as *HSPA1A*, *BMP5*, *NELL2*, *NRK*, and *CXCL13* together with 169 downregulated genes including *GPX3* (Figure 1A). The heatmap data clearly represented the overall expression patterns of the top 10 genes with increased activity and the 10 genes with decreased activity, among which *HSPA1A* ranked in the top four (Figure 1B). Subsequently, the expression of HSPA1A was examined in BPH samples of an integrated dataset, demonstrating significant upregulation compared to normal controls (Figure 1C). Furthermore, Gene Set Enrichment Analysis (GSEA) revealed that upregulation of HSPA1A correlated with the functional pathway including cell cycle, ROS, mitogen‐activated protein kinases (MAPK) signaling pathway, PI3K/AKT signaling pathway and apoptosis (Figure 1D,E). Moreover, single‐cell transcriptome data from dataset GSE172357 were obtained and analyzed for further investigation. A total of 83,451 cells were analyzed from human prostate samples by graph‐based clustering and uniform manifold approximation and projection (UMAP). Additionally, eight major cellular clusters were annotated, including basal cells, luminal cells, fibroblasts, smooth muscle cells, endothelial cells, lymphoid cells, myeloid cells, and granulocytes (Figure 1F). The feature plot directly demonstrated the expression level of HSPA1A in various cell types, with a significant increase in the clusters of hyperplastic prostate cells compared to normal ones (Figure 1G). To our knowledge, basal and luminal cells constituted the most abundant epithelial cells, while fibroblasts, smooth muscle, and endothelial cells were the three most abundant cell types in the stromal compartment. As shown in Figure 1H, HSPA1A was highly expressed in all these prostate cells from BPH samples versus normal samples. Moreover, Gene Set Variation Analysis (GSVA) revealed that HSPA1A upregulation in epithelial cells affected the cell cycle and MAPK pathway, while in stromal cells, notable changes were observed in apoptosis, cell cycle, MAPK pathway, ROS, and PI3K/AKT pathway (Figure S1).

### 2.2 The Expression and Localization of HSPA1A in Human Prostate Tissues and Prostate Cells

**FIGURE 2:**
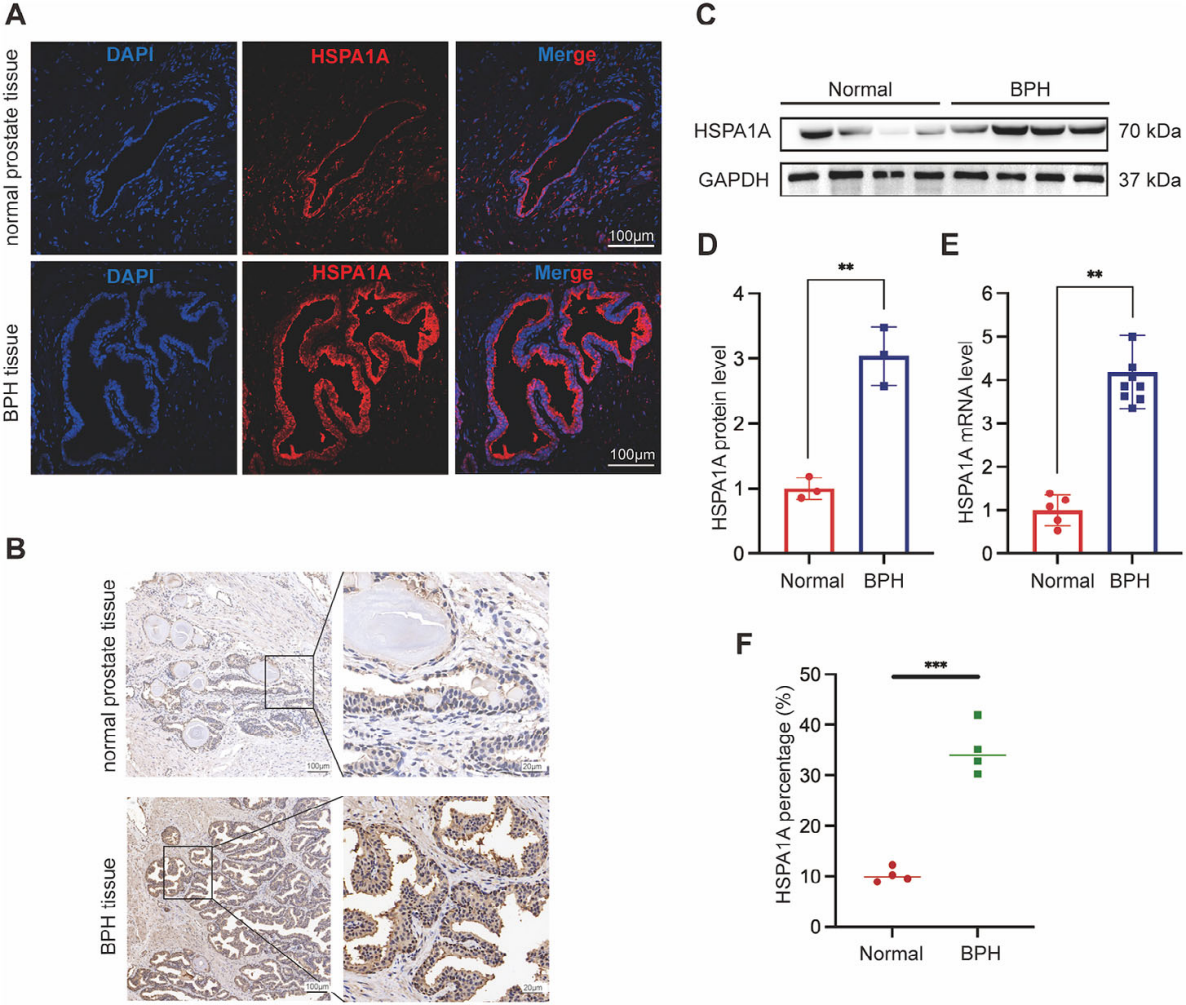
HSPA1A was upregulated in hyperplastic prostate tissues and widely distributed in stromal and epithelial sections of human prostate tissues. (A) Representative immunofluorescence staining for HSPA1A in human BPH tissue and normal prostate tissue. DAPI (blue) indicates nuclear staining, and Cy3‐immunofluorescence (red) indicates HSPA1A protein staining. The scale bars are 100 µm. (B) Representative immunohistochemistry staining for HSPA1A in the human BPH tissue and normal prostate tissue. The scale bars are 100 µm and 20 µm (enlarged). (C, D) Immunoblotting analysis and relative densitometric quantification of HSPA1A in prostate tissues (*n* = 3). (E) The mRNA expression level of HSPA1A in BPH tissues versus normal tissues by qRT‐PCR (*n* = 3). (F) IHC analysis of HSPA1A in prostate tissues (*n* = 4). GAPDH is used as a loading control. **p < 0.01; ***p < 0.001.

Human normal and BPH prostate tissues (*n* = 4, each) were harvested from Zhongnan Hospital of Wuhan University. Immunofluorescence staining revealed the presence of HSPA1A in both the epithelial and stromal compartments of human prostate samples (Figures 2A and S2). Additionally, immunohistochemistry (IHC) staining showed a significant upregulation in the BPH groups compared to the normal ones (Figure 2B,F). Furthermore, the HSPA1A protein level was elevated by as much as threefold compared to normal prostate tissues (Figure 2C,D). Similarly, the mRNA level of HSPA1A exhibited a significant over fourfold increase in BPH samples (Figure 2E).

### 2.3 Silencing of HSPA1A Inhibited Cell Viability but Had No Effect on Cell Cycle in Human Prostate Cells

**FIGURE 3:**
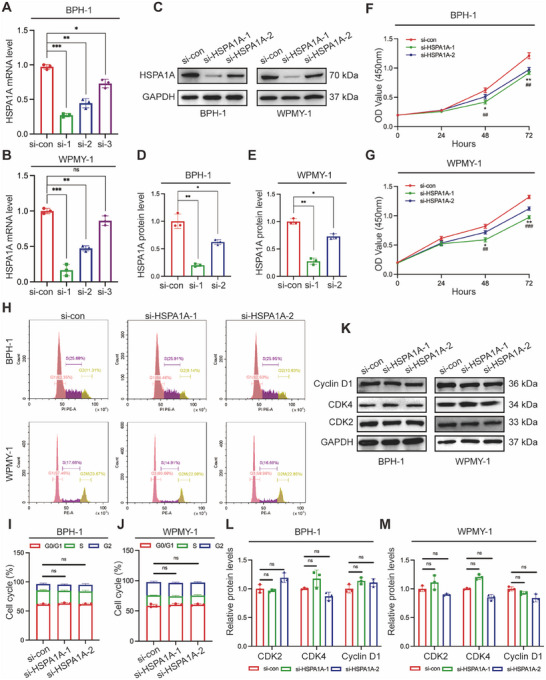
Downregulation of HSPA1A suppressed cell proliferation but did not affect the cell cycle. (A, B) Knockdown efficiency of HSPA1A at the mRNA levels with three different siRNA sequences (si‐HSPA1A‐1, si‐HSPA1A‐2, and si‐HSPA1A‐3) in BPH‐1 and WPMY‐1 cells (*n* = 3). (C) Immunoblotting analysis and (D, E) relative densitometric quantification of HSPA1A in prostate cells with si‐HSPA1A‐1 and si‐HSPA1A‐2 (*n* = 3). (F, G) Cell viability of BPH‐1 and WPMY‐1 cells after knockdown of HSPA1A at different time points by CCK‐8 assay (*n* = 3). (H) Cell cycle for BPH‐1 and WPMY‐1 cells by flow cytometry analysis. The percentages (%) of cell populations at different cell cycle stages are listed within the panels. (I, J) Statistical analysis of cell percentage in each cell cycle phase (*n* = 3). (K) Immunoblotting analysis and (L, M) relative densitometric quantification of protein CDK2, CDK4, and Cyclin D1 in HSPA1A‐silenced prostate cells (*n* = 3). GAPDH is used as a loading control. ns: *p* > 0.05; *p < 0.05; **p < 0.01; ***p < 0.001.

Furthermore, we continued our investigation into the role of HSPA1A in BPH‐1 and WPMY‐1 prostate cells. HSPA1A‐deficient cell models were constructed with the transfection of three different HSPA1A‐specific siRNAs (si‐HSPA1As). All candidates were confirmed to efficiently downregulate HSPA1A at both mRNA and protein expression levels in both cell lines (Figure 3A–E). Si‐HSPA1A‐1 and si‐HSPA1A‐2 were ultimately selected for further experiments due to their stronger inhibitory effects compared to si‐HSPA1A‐3. Notably, HSPA1A silencing resulted in an inhibitory effect on cell viability in BPH‐1 or WPMY‐1 cells after 48 and 72 h (Figure 3F,G). Subsequently, the cell cycle was validated by flow cytometry and immunoblotting assays. There was no significant difference observed in the G0/G1, S, or G2 phases following HSPA1A deficiency either in BPH‐1 or WPMY‐1 cell lines (Figure 3H–J). Similarly, cell cycle‐related proteins such as CDK2, CDK4, or Cyclin D1 remained largely unchanged in both prostate cell lines (Figure 3K–M).

### 2.4 Deficiency of HSPA1A Enhanced Cell Apoptosis, ROS Accumulation, and Activated ERK/JNK Pathway

**FIGURE 4:**
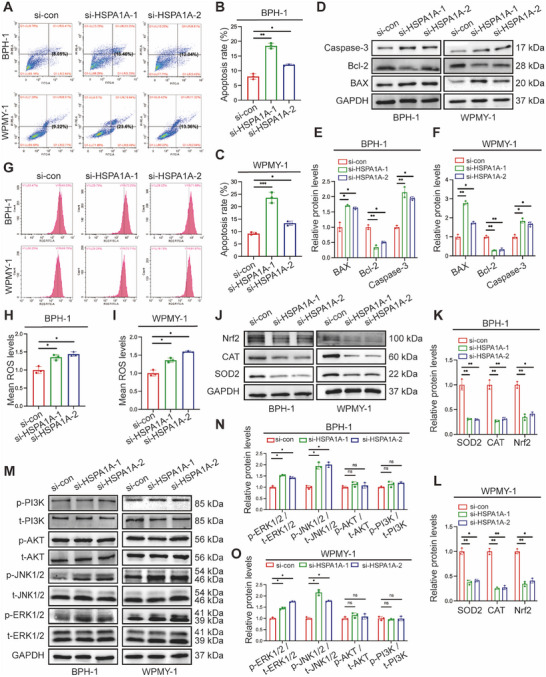
Silencing of HSPA1A promoted apoptosis and ROS production together with activating the ERK/JNK cascade. (A) Flow cytometry analysis of cell apoptosis upon transfection with either control or siRNAs. PI PE‐A in the *y*‐axis stands for the fluorescence intensity of propidine iodide (PI), and FITC‐A in the *x*‐axis stands for the fluorescence intensity of Fluorescein isothiocyanate (FITC) labeled Annexin V. The apoptosis rate is represented by the percentage of Annexin V+/PI+ cells. (B, C) Statistical analysis of apoptosis rate in BPH‐1 and WPMY‐1 cells (*n* = 3). (D) Immunoblotting analysis and (E, F) relative densitometric quantification of protein BAX, Bcl‐2, and Caspase‐3 in HSPA1A‐silenced prostate cells (*n* = 3). (G) Flow cytometry analysis of ROS production in BPH‐1 and WPMY‐1 cells with either control or siRNAs. (H, I) Statistical analysis of mean ROS levels (*n* = 3). (J) Immunoblotting analysis and (K, L) relative densitometric quantification of protein SOD2, CAT, and Nrf2 in HSPA1A‐silenced prostate cells (*n* = 3). (M) Immunoblotting analysis and (N, O) relative densitometric quantification of protein p‐ERK, p‐JNK, p‐AKT, and p‐PI3K in HSPA1A‐silenced prostate cells (*n* = 3). GAPDH is used as a loading control. ns: *p* > 0.05; *p < 0.05; **p < 0.01; ***p < 0.001.

To further investigate the potential role of HSPA1A in cell survival, we evaluated cell apoptosis levels using flow cytometry and related protein markers. Flow cytometry analysis showed silencing HSPA1A remarkably elevated the apoptosis levels compared to controls. Specifically, the proportion of apoptotic BPH‐1 cells increased dramatically from 8.05% to 18.46% and 12.04%, respectively, upon transfection with si‐HSPA1A‐1 and si‐HSPA1A‐2. Consistently, the average apoptotic WPMY‐1 cells increased from 9.22% to 23.6% and 13.36%, respectively, following HSPA1A knockdown (Figure 4A–C). Moreover, Western blotting analysis demonstrated that apoptosis‐associated proteins such as the Bcl‐2‐associated X protein (BAX) and Caspase‐3 were upregulated in the aforementioned groups, while Bcl‐2 completely exhibited the opposite trend (Figure 4D–F). Furthermore, silencing HSPA1A led to a significant increase in ROS accumulation as demonstrated by flow cytometry (Figure 4G–I). Parallelly, the protein levels of ROS‐resistance genes, including superoxide dismutase 2 (SOD2), catalase (CAT), and nuclear factor erythroid 2‐related factor 2 (Nrf2), were all reduced by over half (Figure 4J–L), suggesting that HSPA1A deficiency substantially promoted ROS overproduction. Meanwhile, we detected the protein levels of the MAPK family along with the PI3K/AKT cascade in BPH‐1 or WPMY‐1 cells due to its crucial role in modulating cell proliferation and apoptosis. Interestingly, suppression of HSPA1A led to significant intensification of downstream effectors of the MAPK pathway, including the proportion of phosphorylated ERK1/2 (p‐ERK1/2) and phosphorylated JNK1/2 (p‐JNK1/2), whereas no obvious alteration of phosphorylated AKT (p‐AKT) or phosphorylated PI3K (p‐PI3K) was observed. (Figure 4M‐O).

### 2.5 Upregulation of HSPA1A Promoted Cell Proliferation but Barely Affected Cell Cycle

**FIGURE 5:**
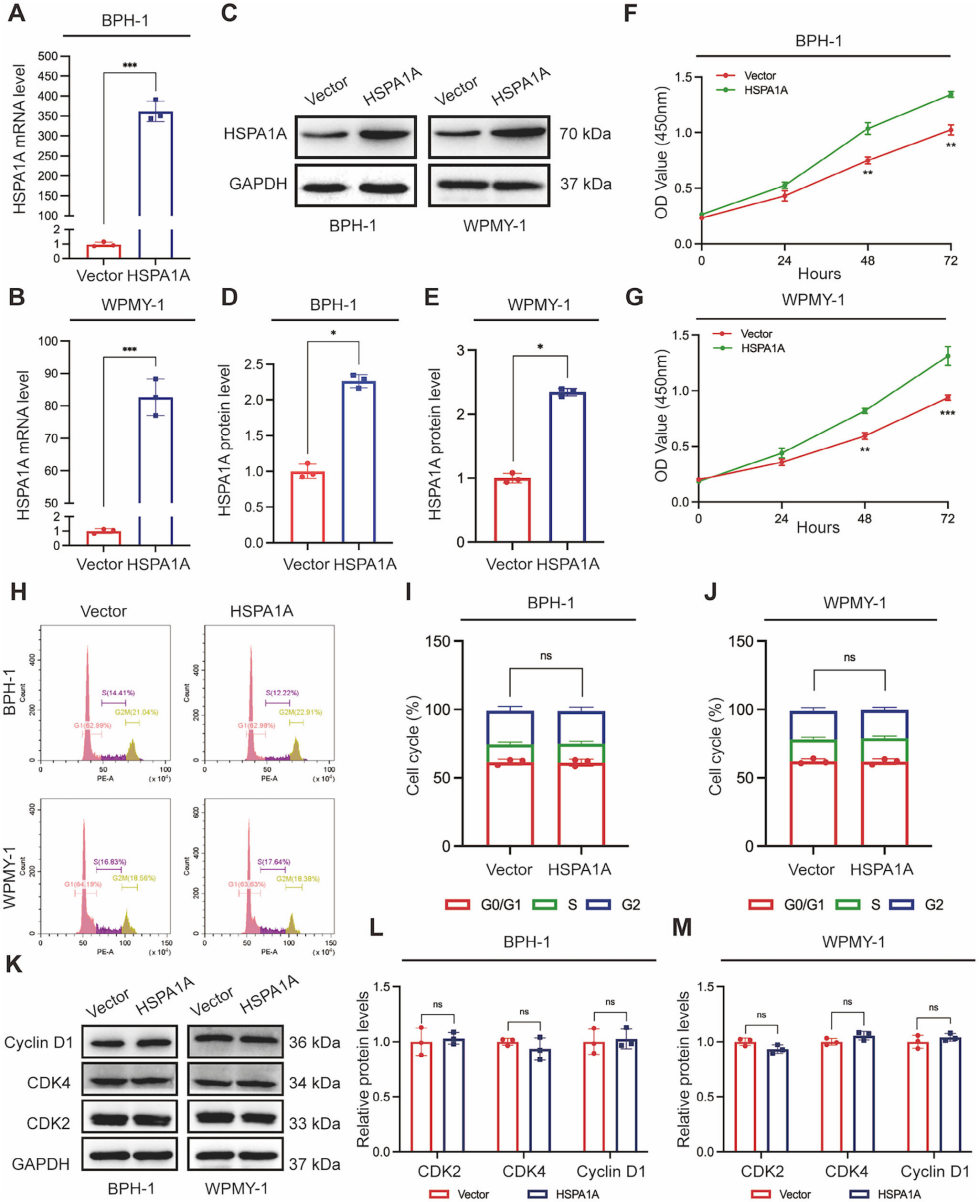
Overexpression of HSPA1A enhanced cell proliferation but did not affect the cell cycle. (A, B) The mRNA level of HSPA1A in BPH‐1 and WPMY‐1 cells transfected with either HSPA1A plasmid or vector (*n* = 3). (C) Immunoblotting analysis and (D, E) relative densitometric quantification of HSPA1A protein expression (*n* = 3). (F, G) Cell viability of both prostate cells after HSPA1A overexpressed at different time points by CCK‐8 assay (*n* = 3). (H) Cell cycle for BPH‐1 and WPMY‐1 cells by flow cytometry analysis. (I, J) Statistical analysis of cell percentage in each cell cycle phase (*n* = 3). (K) Immunoblotting analysis and (L, M) relative densitometric quantification of protein CDK2, CDK4, and Cyclin D1 in HSPA1A overexpressed prostate cells (*n* = 3). GAPDH is used as a loading control. ns: *p* > 0.05; *p < 0.05; **p < 0.01; ***p < 0.001.

Next, we transfected the HSPA1A‐targeted plasmid into BPH‐1 and WPMY‐1 cells so as to upregulate the expression of HSPA1A. Quantitative Real‐Time PCR (qRT‐PCR) and Western blotting analyses revealed significant overexpression of HSPA1A compared to vector control in both prostate cells (Figure 5A–E). Cell Counting Kit‐8 (CCK‐8) analysis demonstrated enhanced proliferation of both cell lines 48 and 72 h post‐transfection with plasmids (Figure 5F,G). However, the flow cytometry assay indicated minimal alterations in cell cycle phases upon HSPA1A upregulation, which were not statistically significant (Figure 5H–J). Simultaneously, the protein markers of the cell cycle, including CDK2, CDK4, and Cyclin D1, showed no significant difference between HSPA1A overexpression and control groups in either cell line (Figure 5K–M).

### 2.6 Overexpression of HSPA1A Attenuated Cell Apoptosis, ROS Deposition, and Deactivated ERK/JNK Pathway

**FIGURE 6:**
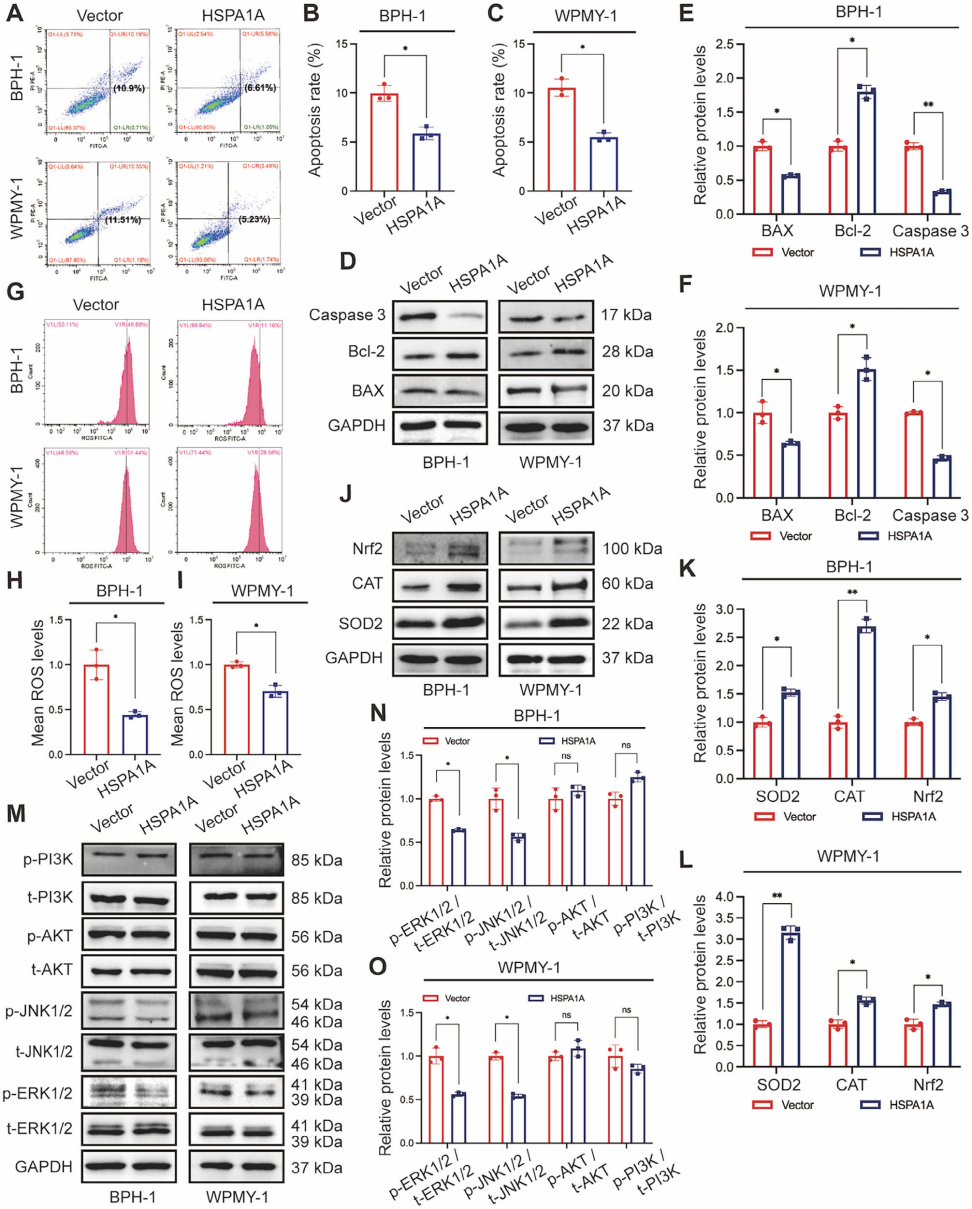
Upregulation of HSPA1A inhibited apoptosis and ROS generation along with deactivating the ERK/JNK cascade. (A) Flow cytometry analysis of cell apoptosis upon transfection with HSPA1A plasmid or vector. (B, C) Statistical analysis of apoptosis rate in BPH‐1 and WPMY‐1 cells (*n* = 3). (D) Immunoblotting analysis and (E, F) relative densitometric quantification of protein BAX, Bcl‐2, and Caspase‐3 in HSPA1A overexpressed prostate cells (*n* = 3). (G) Flow cytometry analysis of ROS production in BPH‐1 and WPMY‐1 cells with either vector or plasmid. (H, I) Statistical analysis of mean ROS levels (*n* = 3). (J) Immunoblotting analysis and (K, L) relative densitometric quantification of protein SOD2, CAT, and Nrf2 in HSPA1A overexpressed prostate cells (*n* = 3). (M) Immunoblotting analysis and (N, O) relative densitometric quantification of protein p‐ERK, p‐JNK, p‐AKT, and p‐PI3K in HSPA1A overexpressed prostate cells (*n* = 3). GAPDH is used as a loading control. ns: *p* > 0.05; *p < 0.05; **p < 0.01; ***p < 0.001.

The functional activity of HSPA1A overexpression was further examined in the context of cell apoptosis and ROS level. The percentage of apoptotic cells showed a significant reduction, dropping from 10.9% to 6.61% for BPH‐1 cells and from 11.51% to 5.23% for WPMY‐1 cells, upon transfection of overexpressed HSPA1A (Figure 6A–C). Similarly, ROS levels significantly descended in the upregulated HSPA1A group (Figure 6D–F). Furthermore, validation via Western blotting confirmed reduced levels of BAX and Caspase‐3, along with increased levels of Bcl‐2 protein (Figure 6G–I). Additionally, the antioxidant enzymes such as SOD2, CAT, and Nrf2 were all downregulated upon HSPA1A overexpression in both prostate cells (Figure 6J–L). Conversely, in contrast to the effects observed with HSPA1A silencing, upregulation of HSPA1A markedly reduced the protein levels of p‐ERK1/2 and p‐JNK1/2. Notably, overexpressed HSPA1A barely altered the relative proportions of p‐AKT or p‐PI3K in total protein (Figure 6M–O).

### 2.7 U0126 Reversed the Impact of HSPA1A Knockdown on Prostate Cells

**FIGURE 7:**
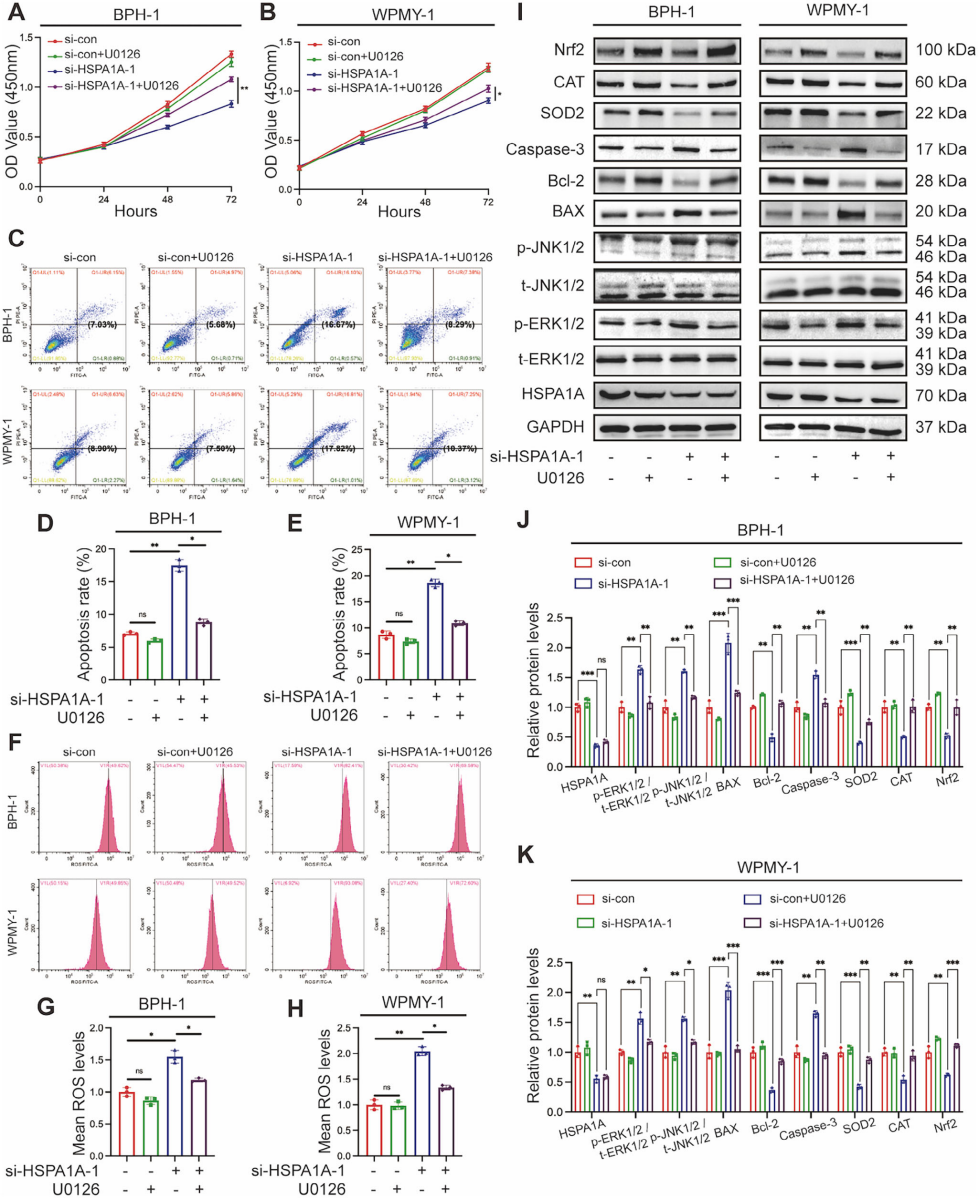
U0126 reversed the effect of HSPA1A knockdown on prostate cells. (A, B) BPH‐1 and WPMY‐1 cells were pretreated with U0126 at 10 µM for 2 h and treated with si‐HSPA1A‐1 for 48 h, compared with control cells. Cell viability of the BPH‐1 and WPMY‐1 cells was analyzed by CCK‐8 assay (*n* = 3). (C) Flow cytometry analysis of cell apoptosis in prostate cells with 48 h si‐HSPA1A‐1 treatment and 2 h U0126 (10 µm) treatment. (D, E) Statistical analysis of apoptosis rate in BPH‐1 and WPMY‐1 cells (*n* = 3). (F) Flow cytometry analysis of ROS production in prostate cells. (G, H) Statistical analysis of mean ROS levels (*n* = 3). (I) Immunoblotting analysis and (J, K) relative densitometric quantification of molecular targets related to apoptosis, ROS, and ERK/JNK pathway in prostate cells with 48 h si‐HSPA1A‐1 treatment and 2 h U0126 (10 µM) treatment (*n* = 3). GAPDH is used as a loading control. ns: *p* > 0.05; *p < 0.05; **p < 0.01; ***p < 0.001.

To determine whether HSPA1A mediated apoptosis and OS through the ERK/JNK pathway, we treated BPH‐1 and WPMY‐1 cells with U0126, a selective MAPK inhibitor, either alone or in combination with si‐HSPA1A‐1. CCK‐8 assay indicated that the suppression of cell viability resulting from si‐HSPA1A‐1 could be significantly rescued by U0126 (Figure 7A,B). Importantly, the impact on apoptosis and ROS exerted by silencing HSPA1A was remarkably reversed by the combined utilization of si‐HSPA1A‐1 and U0126 by flow cytometry assay (Figure 7C–H). We further validate the protein levels by Western blotting, indicating a reduction in p‐ERK1/2, p‐JNK1/2, BAX, and Caspase‐3, along with an augment of SOD2, CAT, and Nrf2, while HSPA1A expression remained invariable (Figure 7I–K).

### 2.8 Inhibition of HSPA1A Alleviated Prostatic Hyperplasia via ERK/JNK Pathway In Vivo

**FIGURE 8:**
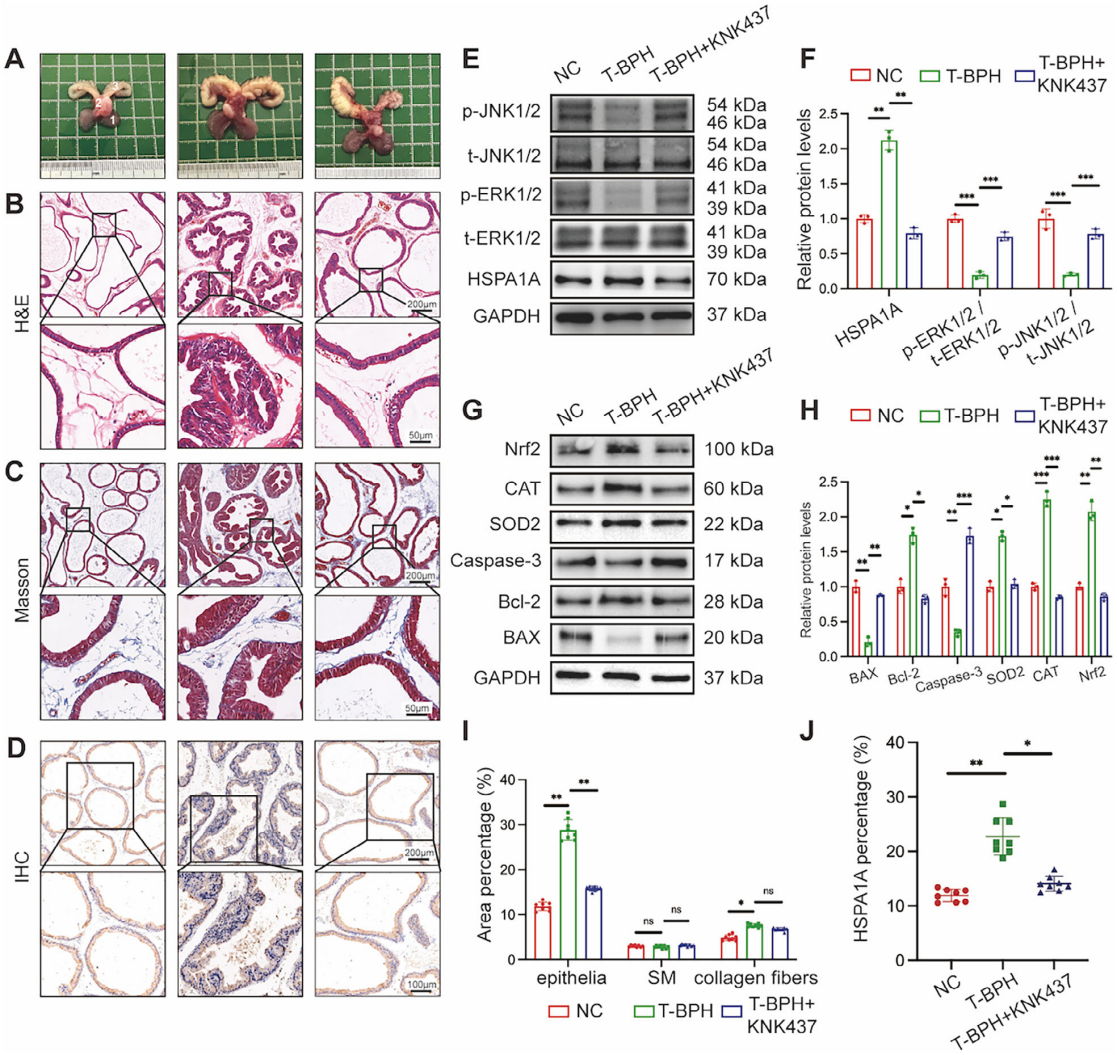
Inhibition of HSPA1A ameliorated prostatic hyperplasia via ERK/JNK cascade in rat models. (A) The rat urogenital tissues from NC, T‐BPH, and T‐BPH + KNK437 rats. (1) ventral prostate, (2) bladder, and (3) seminal vesicle. (B) Representative H&E staining of rat prostates for each treatment group. Scale bars: 200 µm and 50 µm (enlarged). (C) Masson's trichrome staining of rat prostates for each treatment group; prostate epithelial cells were stained auburn, smooth muscle (SM) cells were stained red, and collagen fibers were stained blue. Scale bars: 200 µm and 50 µm (enlarged). (D) Representative IHC images of HSPA1A in each treatment group. Scale bars: 200 µm and 100 µm (enlarged). (E, F) Immunoblotting analysis of HSPA1A and ERK/JNK pathway in each treatment group (*n* = 3). (G, H) Immunoblotting analysis of protein markers related to apoptosis and ROS in each treatment group (*n* = 3). (I) Quantification of Masson's trichrome staining (*n* = 8). (J) The quantitative analysis of HSPA1A expression in each group of rat prostates (*n* = 8). GAPDH is used as a loading control. ns: *p* > 0.05; *p < 0.05; **p < 0.01.

Following our in vitro study, in vivo experiments were further conducted to confirm the function of HSPA1A in the development of BPH. T‐BPH rats were induced by testosterone for 4 weeks, resulting in a dramatic increase in the weight of both the ventral prostate (v.p.) and seminal vesicles (Figure 8A, Table 1). Histologically, Hematoxylin and Eosin (H&E) staining in T‐BPH rats revealed an increased and enlarged epithelial component compared to the normal control (NC) group (Figure 8B). Masson's staining analysis revealed a noticeable normal co‐increase in collagen fiber deposition within the stromal compartment of the T‐BPH group (Figure 8C). In addition, the suppression of HSPA1A by its specific inhibitor KNK437 significantly mitigated BPH progression, leading to the atrophy of glandular epithelium. Interestingly, there was no statistical alteration in the stromal components under the treatment of KNK437 in the T‐BPH model (Figure 8I). IHC staining showed increased HSPA1A expression in rats with testosterone supplementation, predominantly in the epithelium, which was reduced by KNK437 treatment (Figure 8D–J). Consistent with the results in cells, the Western blotting analysis demonstrated KNK437 reversed the downregulations of p‐ERK1/2 and p‐JNK1/2 and the upregulation of HSPA1A in T‐BPH rats to varying degrees (Figure 8E,F), implicating that inhibition of HSPA1A probably ameliorated prostatic hyperplasia through the ERK/JNK pathway. Accordingly, the protein levels of apoptosis and OS were also detected, indicating that BAX, Bcl‐2, Caspase‐3, SOD2, CAT, and Nrf2 recovered to a certain level with significant changes (Figure 8G,H).

**TABLE 1:** Variation of biometric and physiological parameters in different treatment rats.

	Body weight (g)				
Group	Initial	Final	Ventral prostate weight (mg)	Seminal vesicles weight (mg)	Prostate index
NC	224.5 ± 12.8	456.2 ± 22.4	465.2 ± 40.9	892.5 ± 78.5	1 ± 0.2
T‐BPH	230.8 ± 13.6	392.3 ± 19.7^***^	1147.5 ± 96.5^***^	2252.4 ± 199.7	2.9 ± 0.3^***^
T‐BPH + KNK437	233.7 ± 15.2	389.6 ± 23.5^ns^	965 ± 101.2^###^	2693.7 ± 208.2	2.4 ± 0.3^#^

Abbreviations: NC, normal control; T, testosterone.

*** *p* < 0.001 T‐BPH vs. NC.

^ns^ *p* > 0.05 T‐BPH vs. T‐BPH+KNK437.

^#^ *p* < 0.05 T‐BPH vs. T‐BPH+KNK437.

^###^ *p* < 0.001 T‐BPH vs. T‐BPH+KNK437.

### 2.9 Upregulation of HSPA1A Positively Correlated With Several Clinical Items in BPH Patients

**FIGURE 9:**
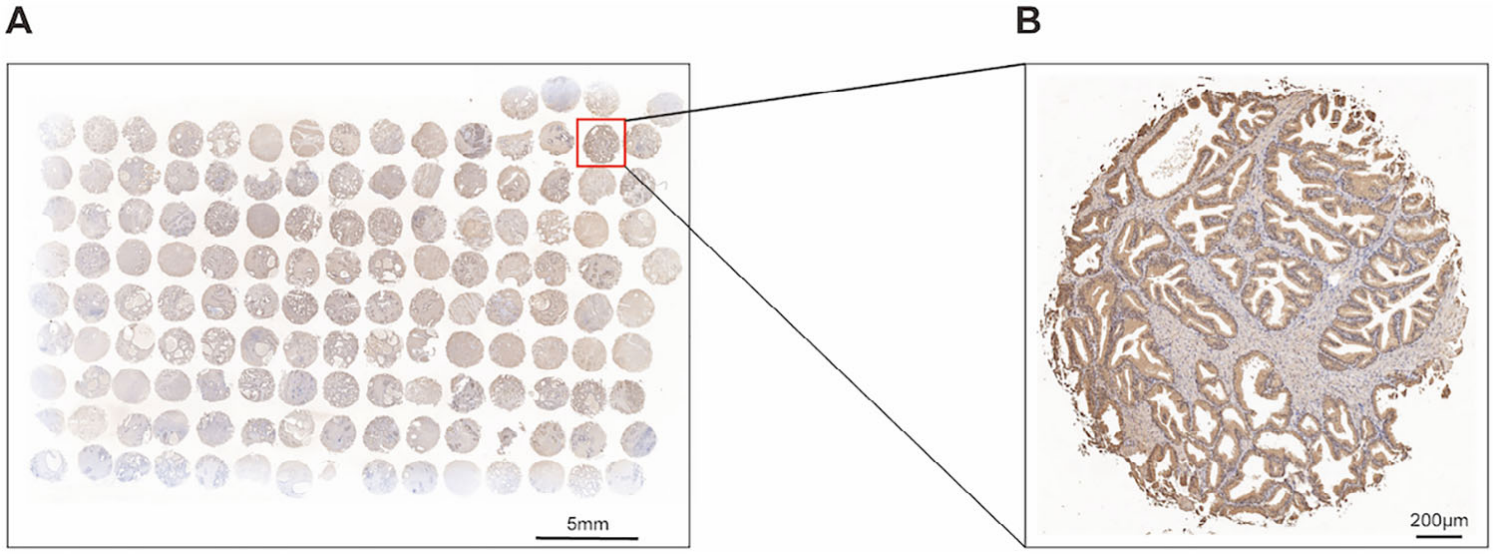
Immunohistochemical analysis of HSPA1A on TMA of human BPH tissues. (A) Immunohistochemical staining of HSPA1A in TMA containing 139 BPH samples. Scale bars: 5 mm. (B) The enlarged image of the tissue sample selected in A. Scale bars: 200 µm.

Ultimately, we utilized 139 human BPH tissues for tissue microarray (TMA) analysis to explore the association between the abundance of HSPA1A and clinical parameters related to BPH. As demonstrated in Figure 9, HSPA1A was expressed in the epithelial compartment as highly as in the stromal compartment of BPH samples. Furthermore, the Pearson correlation analysis showed that HSPA1A was in a positive relationship with prostate volume (PV), total prostate‐specific antigen (tPSA), free prostate‐specific antigen (fPSA), and International Prostate Symptom Score (IPSS). Nevertheless, no significant correlation was observed with any other items (Table 2).

**TABLE 2:** Correlation between the expression level of HSPA1A and clinical parameters of BPH patients.

Items	Correlation index	*p* value
Age (year)	0.1759	0.0892
BMI (kg/m^2^)	0.0972	0.4186
PV (mL)	0.2819^*^	0.0257
tPSA (ng/mL)	0.3618^*^	0.039
fPSA (ng/mL)	0.2362^*^	0.0274
Qmax (m/s)	−0.0977	0.63
RUV (mL)	0.0653	0.7796
IPSS	0.4149^**^	0.0063
Nocturia (times)	−0.2533	0.14

## 3 Discussion

This current study unraveled that HSPA1A was widely distributed in both the stroma and epithelium of human prostate tissues and remarkably upregulated in both compartments of BPH tissues. Through in vitro and in vivo experiments, we manifested that administration of HSPA1A could attenuate apoptosis and OS while facilitating cell proliferation by suppressing the phosphorylation and activation of ERK1/2 and JNK1/2. Collectively, our novel findings implicated a pivotal role for HSPA1A during the period of BPH progress.

Although BPH was widely acknowledged as a disorder arising from aging and dysfunctional testis, the in‐depth mechanism of BPH/LUTS remained undefined. The lack of a precise therapeutic target was a prominent obstacle for BPH treatment [31]. By employing an integrated analysis of two bulk RNA‐seq datasets (GSE119195 and GSE132714), we identified numerous DEGs between human hyperplastic and normal prostate tissues. Gene *HSPA1A* ranked among the top four highly expressed DEGs, including *RN7SK*, *RN7SL4P*, and *BMP5*, in BPH tissues. Gene *RN7SK* and *RN7SL4P*, belonging to the highly conserved noncoding RNA family [32], were excluded in our current study. Moreover, BMP5, as an important therapeutic target for BPH, was identified in our previous study [11]. Thus, HSPA1A greatly attracted our attention for further investigation. Additionally, single‐cell transcriptome analysis showed an apparent upregulation of HSPA1A in human hyperplastic cells including basal, luminal, fibroblast, smooth muscle, and endothelial cells compared to normal prostatic cells. Therefore, it is imperative to investigate the role of HSPA1A in BPH pathogenesis.

HSPA1A, a stress‐inducible protein of the HSP70 superfamily, was abundantly expressed in various organs such as the adrenal gland, lung, liver, and prostate, according to an analysis of the human tissue‐specific expression [33]. Early in 2004, Kramer et al. reported that HSPA1A was relatively upregulated in human BPH specimens [34]. Our findings further demonstrated that HSPA1A was highly expressed in human prostate tissues and equivalently upregulated in the epithelium and stroma of BPH tissues. However, the mechanism underlying the upregulation of HSPA1A in BPH remained unveiled. Recent research has highlighted the significant roles of chronic inflammation and OS in the progression of BPH [35, 36]. Chronic prostatic inflammation, as a key factor in the pathogenesis of BPH, was associated with elevated prostate symptom scores and increased PV [37]. Additionally, OS‐mediated pathways and excessive ROS contributed to the overgrowth of the prostate [38]. Similarly, it was found that patients with nonalcoholic steatohepatitis and chronic hepatitis C infection manifested elevated HSPA1A levels, which increased with the expansion of hepatic inflammation [29]. Also, in inflammatory cells, HSPA1A was upregulated by interacting with Nrf‐2 upon exposure to OS [39]. Based on these studies, we inferred that upregulation of HSPA1A might result from chronic inflammation and OS in the pathogenesis of BPH. Moreover, HSPA1A was localized in both epithelial cell lines and stromal ones (BPH‐1 and WPMY‐1). This finding was slightly inconsistent with another study performed in dogs, which revealed that HSPA1A was localized only in the epithelial basal cells by IHC staining [40]. We hypothesized that this contradiction regarding localization might be attributed to the fact that the canine prostate lacked zonal differentiation observed in human prostates, had much more prominent acini, and comprised less stromal tissue. Similarly, our rat models exhibited a more dramatic alteration in HSPA1A expression in the epithelial compartment than in the stroma. The localization and expression level of HSPA1A in different animal models warranted further investigation.

As a cytoprotective modulator, HSPA1A was found to suppress cell apoptosis and promote cell proliferation in diverse tissues [41]. Previous studies revealed that extracellular HSPA1A could promote the growth of hepatocarcinoma by augmenting cell proliferation and conferring apoptosis resistance [42]. Additionally, HSPA1A inhibited apoptosis and exerted neuroprotective effects in spinal cord injury [25]. In our present study, GSEA analysis indicated that high expression of HSPA1A correlated with cell growth and apoptosis. Meanwhile, HSPA1A knockdown in BPH‐1 and WPMY‐1 cells resulted in a significant increase in apoptosis rate as well as a reduction in cell viability, consistent with the aforementioned studies. The dysregulation between the proliferation and apoptosis of cells was considered a crucial pathogenic mechanism in BPH. Upon exposure to various endogenous or exogenous stimuli, the BAX is recruited to initiate apoptosis [43]. This results in the formation of pores in the mitochondrial membrane, ultimately leading to the release of cytochrome c, a well‐conserved electron‐transport protein and a component of the respiratory chain localized in the mitochondrial intermembrane space, into the cytoplasm. Cytochrome c then triggers Caspase‐9, which subsequently activates Caspase‐3 to induce apoptosis [44]. To regulate the equilibrium between cell death and survival, the Bcl‐2 protein functions as an antiapoptotic factor by inhibiting the activation of BAX [45]. As demonstrated in our study, administration of HSPA1A in vitro could dramatically alter the protein levels of BAX, Bcl‐2, and Caspase‐3, corresponding with its pro‐survival and apoptosis‐resistance role in both prostatic cells.

The cell cycle plays a crucial role in modulating the homeostasis between cell proliferation and apoptosis [46]. Cyclin‐dependent kinases (CDKs) comprise a group of proteins that regulate the cell cycle progression. Among them, CDK2 and CDK4 were essential in the transition from the G1 to the S phase by forming the complex with Cyclin Ds such as CDK2/4‐Cyclin D1 [47]. As an important biological process, the cell cycle was also related to the upregulation of HSPA1A by GSEA enrichment analysis. Nevertheless, our subsequent experiments demonstrated no significant effect on the cell cycle when HSPA1A was either knocked down or overexpressed in prostate cell models. Therefore, we speculated that the inhibitory effect of HSPA1A on apoptosis in prostate cells might not be associated with the regulation of the cell cycle.

ROS is known as a potential contributor to a plethora of chronic diseases due to its ability to alter gene expression, induce gene mutations, and increase cellular mortality [48, 49]. At a fundamental level, a certain level of OS was essential for normal cell functions and signaling. However, excessive production of ROS could hinder cell proliferation and facilitate apoptosis [50]. Existing evidence suggested that overexpression of HSPA1A could protect against liver injury via attenuation of hepatocellular death and OS [29]. Similarly, our bioinformatic analysis revealed that ROS was a significant enrichment pathway when HSPA1A was highly expressed. Moreover, ROS accumulation was considerably increased in HSPA1A‐silenced BPH‐1 or WPMY‐1 cell lines, while it was reduced in HSPA1A‐overexpressed cell models. In addition, the antioxidant enzymes, including SOD2, CAT, and Nrf2, exhibited changes inversely proportional to the ROS levels. Based on these findings, we inferred that upregulated HSPA1A could augment the expression of antioxidant proteins to defend against excessive ROS production and reduce oxidative damage, thereby promoting cell viability and suppressing apoptosis.

Building upon all the observations above, we delved deeper into the potential regulatory mechanisms governing cell proliferation, apoptosis, and OS. The MAPK signaling pathway was involved in a wide range of pathophysiological processes, varying from cell survival, differentiation, apoptosis, autophagy, and inflammation [51, 52, 53, 54]. Typically, MAPK cascades were categorized into four branches: The ERK1/2 cascade, or classical pathway, ERK5, c‐Jun N‐terminal kinase1/2 (JNK1/2), together with p38 pathway [55]. Indeed, in our previous studies, the MAPK pathway was identified as correlating with the progression of BPH. Liu et al. suggested that depletion of NELL2 could induce the apoptosis of prostate cells via activating the ERK1/2 cascade [12]. Furthermore, Li et al. elucidated that coadministration of H_2_O_2_ and silencing PAGE4 could activate JNK1/2 and inhibit ERK1/2, thereby enhancing cell survival and decreasing apoptosis in prostate cells [56]. As previously noted, our bulk RNA‐seq analysis revealed that the MAPK signaling pathway ranked first among potent functional pathways, accompanied by upregulation of HSPA1A. In vitro studies further validated that deficiency of HSPA1A triggered the phosphorylation of ERK1/2 and JNK1/2, while upregulated HSPA1A caused the inactivation of phosphorylated ERK1/2 and JNK1/2 (p‐ERK1/2 and p‐JNK1/2). In this context, we suspected whether HSPA1A could modulate cell proliferation, apoptosis, and OS by deactivating the ERK/JNK pathway. To verify our hypothesis, U0126, a competitive and selective inhibitor of MAPK cascades was added to the HSPA1A silenced cell models as well as negative controls [57]. We observed that U0126 combined with HSPA1A deficiency significantly reduced the protein levels of p‐ERK1/2 and p‐JNK1/2. Meanwhile, the suppression of cell viability caused by HSPA1A depletion was markedly rescued by U0126. Regarding apoptosis and OS, U0126 supplementation could efficiently counteract the elevated levels of apoptosis and ROS. Notably, the protein expression level of HSPA1A showed no alteration by U0126. Given that HSPA1A did not serve as a kinase, we inferred that it might adjust the MAPK signaling cascades indirectly. Hao et al. found that overexpressed HSPA1A could inhibit the activation of JNK to alleviate the effect of Baicalin on COPD [23]. Additionally, Kim et al. established a *Hspa1a^−/−^* mouse model, revealing that deletion of HSPA1A might induce cardiac dysfunction and the development of cardiac hypertrophy through the activation of JNK and ERK [58]. Furthermore, a checkpoint named BPGAP1 (a BCH domain‐containing, Cdc42GAP‐like Rho GTPase‐activating protein) was observed to facilitate JNK to work in concert with ERK in regulating cell proliferation [59]. Based on these findings, we assumed that HSPA1A could enhance cell proliferation and attenuate cell apoptosis and OS by impeding the ERK/JNK pathway in the development of BPH. Finally, we transitioned from an in vitro study to an in vivo experiment. In our T‐BPH rat model, we observed a significant increase in epithelial compartments, accompanied by a slight increase in stroma. KNK437, a targeted inhibitor of HSPs, was reported to effectively reduce the expression of HSPA1A in numerous studies [60, 61, 62]. Intraprostatic injection of KNK437 exhibited therapeutic effects on T‐BPH rats and reversed expression levels of proteins concerning apoptosis, OS, and the ERK/JNK pathway.

Interestingly, TMA analysis confirmed the upregulation of HSPA1A in BPH tissues and revealed its correlation with clinical parameters such as PV, tPSA, fPSA, and IPSS. BPH was characterized by uncontrolled proliferation of epithelial and stromal cells, which resulted in the enlargement of PV [3]. Herein, upregulated HSPA1A was identified to promote the proliferation of epithelial prostate cells, which might account for the positive correlation between HSPA1A expression and PV. PSA levels and IPSS were commonly used as initial screening tools for BPH [63, 64]. It was widely acknowledged that PSA level correlated with the proliferation of epithelial prostate cells. In our study, HSPA1A was found to be upregulated in both the epithelial and stromal compartments of BPH tissues, promoting cell proliferation and inhibiting apoptosis. This upregulation might explain the positive correlation with PSA levels. Furthermore, Park et al. demonstrated a significant correlation between PSA and IPSS [65]. Based on these findings, we inferred that the upregulation of HSPA1A might contribute to LUTS in BPH patients. However, HSPA1A upregulation showed no significant correlation with age (correlation index = 0.1759, *p* = 0.0892). This discrepancy might result from the limited quantities of BPH specimens, which possibly obscured the correlation.

However, some limitations still exist in this study. First of all, since there was a significant age difference between the normal prostate and BPH samples (23.25 ± 4.41 years vs. 68.46 ± 10.06 years), the upregulation of HSPA1A identified in the present study may potentially result from age, instead of BPH itself. However, Luo et al, [66] collected normal prostate samples from histologically normal regions within the radical prostatectomy specimens. Their study made a comparison of gene expression profiles between normal prostate specimens from individuals aged ≥ 59 years and BPH specimens from individuals aged ≤ 59 years. Despite the age difference (52.5 vs. 70.6, normal vs. BPH), they observed similar gene expression alterations, suggesting that age may not constitute an independent variable influencing the outcomes of our study. In fact, around half of men aged 50 and above showed pathological signs of BPH, and this rate rose to more than 80% as men entered their 80s or older. Consequently, it was challenging to obtain healthy prostate samples from men over 70 years of age in the current clinical setting. Second, HSPA1A upregulation showed no significant correlation with age (correlation index = 0.1759, *p* = 0.0892). This discrepancy might result from the limited quantities of BPH specimens, which possibly obscured the correlation. Therefore, a cohort study demonstrating the alterations of HSPA1A over time with BPH progression will be conducted in our future work. Third, since the expression of HSPA1A could not be completely depleted by KNK437 In vivo, a targeted inhibitor of HSPs, the *Hspa1a^−/−^* mouse model, was required to further determine its role in the development of BPH.

In summary, this study highlights the prominent role of HSPA1A in orchestrating cell proliferation, apoptosis, and OS during the progression of BPH. We proved that upregulated HSPA1A could exert an inhibitory impact on cell apoptosis and OS while promoting cell proliferation via the inactivation of the ERK/JNK pathway in vitro and in vivo. Our research suggested that the inhibition of HSPA1A might present a viable therapeutic strategy for BPH.

## 4 Materials and methods

### 4.1 Bulk RNA‐Seq and Single‐Cell Transcriptome Analysis

Raw gene expression profiles, including GSE119195 and GSE132714, were collected from the GEO database (http://www.ncbi.nlm.nih.gov/geo/). R software (version 4.3.2), together with corresponding packages, was utilized to analyze these datasets. The expression matrices of GSE119195 and GSE132714 were normalized and integrated. By utilizing the R package “limma” (version 3.58.1), the combined dataset was processed to identify DEGs, and the threshold criteria for which were set at p < 0.05 and |log_2_FC| > 1. Following this, to visualize the expression data of DEGs, separate volcano images and heatmaps were constructed. According to the median expression level of the HSPA1A gene, the BPH samples of the integrated dataset were divided into two groups. Then REACTOME (https://curator.reactome.org/) and Kyoto Encyclopedia of Genes and Genomes (KEGG) (https://www.genome.jp/kegg/) pathways between the high and low expression groups were distinctly analyzed by utilizing the GSEA. R packages “orq.Hs.eq.db” (version 3.18.0) and “clusterProfiler” (version 3.14.3) were employed for GSEA analysis, and *p* < 0.05, FDR < 0.25 were deemed to be statistically significant [67]. The single‐cell transcriptome data of GSE172357 and annotated data were downloaded from CZ CELL×GENE Discover (https://cellxgene.cziscience.com/). R package “Seurat” (version 4.4) was used to analyze the expression of HSPA1A in various cell types [68]. Based on the median expression of HSPA1A, epithelial cells and stromal cells were separated into two distinct cell subsets. The reference gene set was retrieved by the R package “msigdbr” (version 7.5.1), and then GSVA was conducted on different subsets to analyze functional differences [69].

### 4.2 Human Prostate Tissue Samples

Human prostate tissues were obtained from four young, healthy, brain‐dead men, over 18 years old and under 35 years old (mean age, 23.25 ± 4.41 years), who underwent organ donation in Zhongnan Hospital of Wuhan University as normal controls (from April 2023 to May 2023), with pathological examination revealing no hyperplasia. All samples were granted for research use by donors or their close relatives with an informed consent form on organ donation signed prior to donation. The prostate specimens of 139 BPH patients (mean age, 68.46 ± 10.06 years) together with clinical data from those who underwent transurethral resection of the prostate with indications for BPH surgery in the Department of Urology, Zhongnan Hospital of Wuhan University, were obtained (from October 2022 to September 2023). The diagnosis of BPH was verified through postoperative pathological analysis, which was reviewed and confirmed by two independent pathologists. Patients with a history of Type 2 diabetes, Alzheimer's disease, various cancers, chronic obstructive pulmonary disease, or prostatitis/urinary tract infection, and a history of oral medicine such as antidiabetic drugs and statins possibly affecting the expression level of HSPA1A were excluded in our study [20–23, 70]. Prostate tissue samples were separated into two parts: one portion was preserved in one liquefied nitrogen for immunoblotting and qRT‐PCR analysis, while the other was fixed in 4% PFA for histology experiments. All human tissue samples were gathered and utilized following approved Ethics Committee guidelines as well as the principles outlined in the Declaration of Helsinki.

### 4.3 Animal Models

A total of 24 male Sprague Dawley rats (6 weeks old, weighing 200–250 g) were obtained and randomly assigned to three experimental groups (*n* = 8 each): (1) normal control group (NC), which received subcutaneous (s.c.) injections of corn oil (MedChemExpress, China); (2) testosterone‐induced BPH model group (T‐BPH), which received daily s.c. injections of testosterone (Sigma‐Aldrich, USA) at a dose of 2 mg/day combined with corn oil; and (3) T‐BPH + KNK437 group, which received the same testosterone/corn oil treatment as the T‐BPH group, plus KNK437 (MedChemExpress, China) dissolved in dimethyl sulfoxide (DMSO), injected directly into the v.p. KNK437 was administered at a concentration of 10 nM in a 50 µL volume of sterile normal saline, using a 30‐gauge needle to inject both the right and left ventral lobes of the prostate. For the NC and T‐BPH groups, the injection consisted of 50 µL of sterile saline with a similar amount of DMSO. All procedures followed the protocols described previously [71]. The animal experiments were conducted at the Animal Center of Zhongnan Hospital, Wuhan University, and were approved by the institution's Medical Ethics Committee prior to initiation.

### 4.4 TMA Analysis

A comprehensive summary of the clinical features of 139 BPH patients is presented in Table 3. Tissue samples from each of the 139 patient cases were fixed and made into donor wax block sections, which were then subjected to the extraction of a 1.5 mm diameter core to generate TMA. Additionally, these cores were sliced into 4 µm for subsequent staining procedures. A correlation analysis was performed to examine the relationship between the expression levels of HSPA1A and various clinical characteristics. For variables that followed a normal distribution, Pearson's correlation method was used, while non‐normally distributed variables were analyzed using Spearman's correlation. Two‐tailed *p* values were calculated, with a threshold of 0.05 set for statistical significance. Furthermore, stratified analyses were carried out based on patient age groups: 50–60 years, 60–70 years, 70–80 years, and those over 80 years.

**TABLE 3:** Clinical information of 139 BPH patients.

Items	Mean value	SD
Age (year)	68.46	10.06
BMI (kg/m^2^)	23.31	3.10
PV (mL)	52.49	14.57
tPSA (ng/mL)	4.80	2.58
fPSA (ng/mL)	1.33	0.29
Qmax (mL/s)	11.02	8.91
RUL (mL)	93.95	20.68
IPSS	23.13	8.49
Nocturia (times)	4.40	1.79

### 4.5 IHC, H&E Staining, and Masson's Trichrome Staining

The sections were first embedded with paraffin, and then cleared of paraffin using xylene, followed by dehydration through a series of alcohol solutions: anhydrous ethanol, then 95% ethanol, and finally 75% ethanol. For antigen retrieval, these sections were immersed in 10 mM sodium citrate buffer (pH 6.0) and then boiled for 2 min. To deactivate endogenous peroxidase, the sections were exposed to a 3% hydrogen peroxide solution for 10 min. Nonspecific binding sites were blocked by incubating the sections in 15% normal goat serum for 15 min. The sections were then sequentially incubated with primary antibodies (Table S1) at 4°C in a humidified environment, followed by incubation with a secondary antibody at room temperature. Peroxidase activity was visualized with 3,3′‐diaminobenzidine tetrahydrochloride. PBS was used in place of antibodies for the negative controls. To observe and capture the images, an Olympus‐DP72 light microscope was utilized (Olympus, Japan).

H&E staining and Masson's trichrome staining were performed as previously reported [72].

### 4.6 Tissue Immunofluorescence Staining

Prostate tissue samples from humans were sliced into 10 µm‐thick sections, thawed, and placed onto glass slides using a cryostat (Leica CM 1850, Wetzlar, Germany). The sections were allowed to air‐dry and then fixed in ice‐cold acetone for 10 min. Afterward, the slides were washed with PBS and incubated for 2 h in a PBS solution containing 0.2% Triton X‐100 and 0.1% bovine serum albumin. They were then incubated overnight with primary antibodies (Table S1). To detect the primary antibodies, secondary antibodies (Table S2) were applied and then visualized with Cy3‐conjugated goat anti‐rabbit IgG. The nucleus was stained with DAPI, and imaging was performed using a laser scanning confocal microscope (Olympus, Japan).

### 4.7 Cell Culture and Transfection

Human Prostatic cell lines, including BPH‐1 epithelial cell line and WPMY‐1 human normal prostatic stromal cell line, were acquired from Procell (Wuhan, China) and Stem Cell Bank of the Chinese Academy of Sciences (Shanghai, China), respectively. The siRNAs targeting HSPA1A were procured from Genepharma (Suzhou, China). For the overexpression of HSPA1A, a plasmid specifically targeting HSPA1A was synthesized by the Miaoling plasmid platform (Wuhan, China), with an empty plasmid (Vector) serving as the control. These siRNAs or plasmids were separately added to Opti‐MEM reduced serum medium and then combined with Lipofectamine 2000 (Invitrogen, USA) following the manufacturer's protocol. The sequences of the HSPA1A siRNAs are provided in Table S3.

### 4.8 CCK‐8 Assay

BPH‐1 and WPMY‐1 cells were plated in 6‐well plates and transfected with or without si‐HSPA1A‐1/2. After transfection, the cells were cultured for 0, 24, 48, or 72 h. Cell viability was detected using CCK‐8 (C0005, TargetMol) and 5‐ethynyl‐20‐deoxyuridine (EdU) (C10310‐1, RiBoBio) according to the manufacturer's guidelines.

### 4.9 Flow Cytometry Analyses of Cell Cycle, Apoptosis, and ROS

Cells were harvested and washed three times using PBS. To analyze the cell cycle, the cells were resuspended in 1 mL of DNA staining solution, followed by the addition of 10 µL of propidium iodide (PI) solution (Multi‐Sciences Biotech, China). The cells were incubated in the dark for at least 30 min. For apoptosis assessment, the cells were resuspended in 100 µL of 1× Annexin V binding buffer, and then 5 µL of Annexin V‐FITC and 5 µL of PI solution (Sungene Biotech, China) were added sequentially. After 30 min of incubation, 400 µL of 1× Annexin V binding buffer was added. Parameter settings were adjusted according to the specific instructions for each experiment. For ROS analysis, the cells were resuspended in 1 mL of FBS‐free medium, followed by the addition of 1 µL of DCFH‐DA (Sigma, D6883). After a 30‐minute incubation, the cells were centrifuged and resuspended in 1 mL of FBS‐free medium. Flow cytometry (Beckman, USA) was used to analyze each sample.

### 4.10 Total RNA Extraction, Reverse Transcription, and qRT‐PCR

RNA was extracted in accordance with the guidelines provided by the manufacturer for the HiPure Total RNA Mini Kit (Magen, China). For qRT‐PCR, the SYBR Green Fast qPCR Mix (ABclonal, China) was used. The primer sequences are listed in Table S4.

### 4.11 Western Blotting Analysis

Cells and tissues were subjected to lysis in RIPA buffer (Beyotime, China) supplemented with protease and phosphatase inhibitors. An equal quantity of cell lysates was then resolved via SDS‐PAGE. Antibodies are listed in Tables S1 and S2.

### 4.12 Statistical Analysis

The experiments were conducted a minimum of three times. Statistical analysis was performed using SPSS 22.0 software (IBM, USA). Results are presented as the mean ± standard deviation (S.D.). To compare multiple groups, one‐way ANOVA was applied, while Student's *t*‐test was used for comparisons between two groups. Pearson's correlation coefficient was employed for correlation analysis. A *p* value of less than 0.05 was considered statistically significant.

## Author Contributions

Huan Liu, Yongying Zhou, Zhen Wang, Daoquan Liu and Yan Li contributed equally to this work. Huan Liu, Yongying Zhou, Zhen Wang and Xinhua Zhang designed the experiment. Huan Liu drafted the initial manuscript. Huan Liu, Yongying Zhou and Zhen Wang carried out the principal experiments including molecular and animal experiments. Daoquan Liu, Huan Lai, Yuming Guo and Ping Chen assisted in data analysis and specimen collection. Yan Li, Jizhang Qiu, Shidong Shan, Feng Guo and Guang Zeng were involved in the animal experimentation process. Xinhua Zhang and Michael E. DiSanto made critical revisions to the manuscript. Huan Liu and Xinhua Zhang contributed to the writing of the manuscript. All authors have read and approved the final manuscript.

## Ethics Statement

This research was conducted in strict accordance with the ethical principles outlined in the Declaration of Helsinki. The acquisition and handling of all human samples adhered to ethical standards established by the Ethics Committee at Zhongnan Hospital of Wuhan University (No. 2021038). Written informed consent was obtained from all participants. Additionally, all animal‐related procedures were approved by the Experimental Animal Welfare Ethics Committee, Zhongnan Hospital of Wuhan University (No. ZN2023116).

## Conflicts of Interest

The authors declare no conflicts of interest.

## Supporting information



Supporting Information

## Data Availability

The data used to support the findings of this study are available from the corresponding author upon reasonable request.
