# Supplementary material for: Heat Shock Protein Family A Member 1A Attenuates Apoptosis and Oxidative Stress via ERK/JNK Pathway in Hyperplastic Prostate
Source: MedComm (2020). 2025 Mar 10;6(3):e70129. doi: 10.1002/mco2.70129 (PMC11891570; doi:10.1002/mco2.70129)
Supplement: Supplementary file 1 — Supporting Information [file MCO2-6-e70129-s001.docx]

**Heat shock protein family A member 1A attenuates apoptosis and oxidative stress via ERK/JNK pathway in hyperplastic prostate**

Huan Liu^1#^, Yongying Zhou^1#^, Zhen Wang^2#^, Daoquan Liu^3#^, Yan Li^1#^, Huan Lai^1^, Jizhang Qiu^1^, Shidong Shan^1^, Feng Guo^1^, Ping Chen^1^, Yuming Guo^1^, Guang Zeng^1^, Michael. E. DiSanto^4^, Xinhua Zhang^1*^

^1^Department of Urology, Zhongnan Hospital of Wuhan University, Wuhan 430071, China

^2^Department of Urology, Ningbo Medical Center LiHuiLi Hospital of Ningbo University, Ningbo 315040, China

^3^Department of Thoracic Surgery, Zhongnan Hospital of Wuhan University, Wuhan 430071, China

^4^Department of Surgery and Biomedical Sciences, Cooper Medical School of Rowan University, Camden, NJ, USA

^#^These authors have contributed equally to this work.

*Corresponding Author Address

Dr. Xinhua Zhang MD, Ph.D.

^1^Department of Urology, Zhongnan Hospital of Wuhan University, 169 Donghu Road, Wuhan 430071, P.R. China

Tel: 18062535196

Email: zhangxinhuad@163.com

**Supplementary Methods:**

**Cell immunofluorescence staining**

For cell immunofluorescence microscopy, cells were seeded on 12 mm coverslips and washed with ice-cold PBS. The coverslips were then fixed with 4% paraformaldehyde (PFA) for 30 min, followed by 0.1% Triton X-100 incubation, and then blocked in goat serum for 30 min at room temperature. Afterward, they were incubated with primary antibody (**Table S1**) at room temperature for 2 h, washed with PBS, and incubated with Cy3- or FITC-labeled secondary antibody (**Table S2**) for 1 h. Nuclei were labeled with DAPI (4ʹ,6-diamidino2-phenylindole) (2 μg/ml). Visualization was done with a laser scanning confocal microscope (Olympus, Japan).

**Supplementary Figures:**


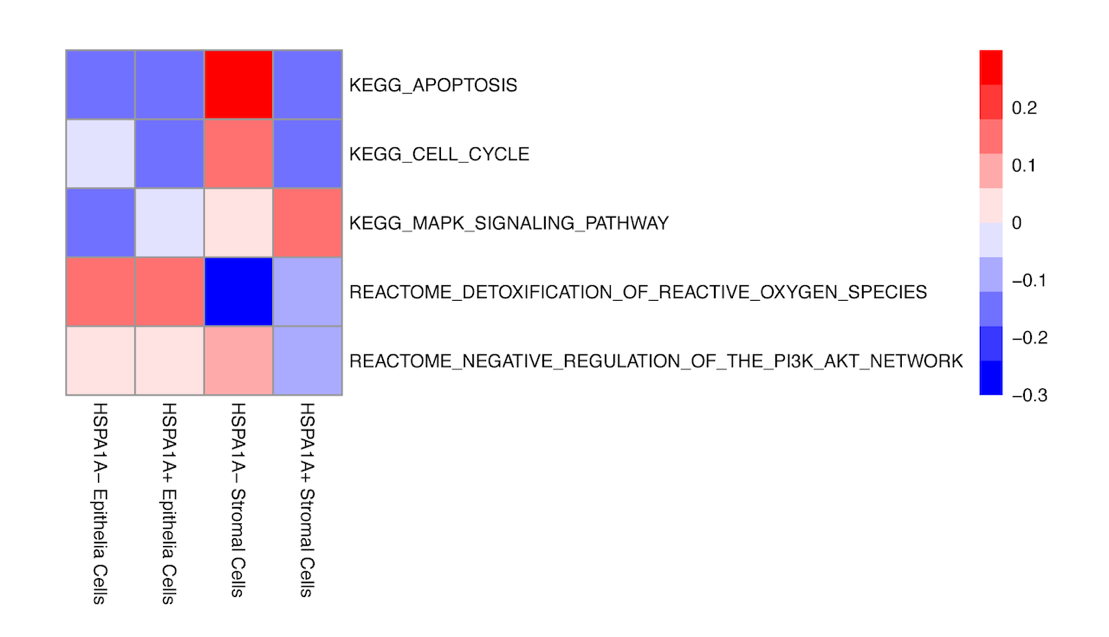


**Figure S1. GSVA analysis of HSPA1A enrichment in different pathways in epithelial and stromal cells.**


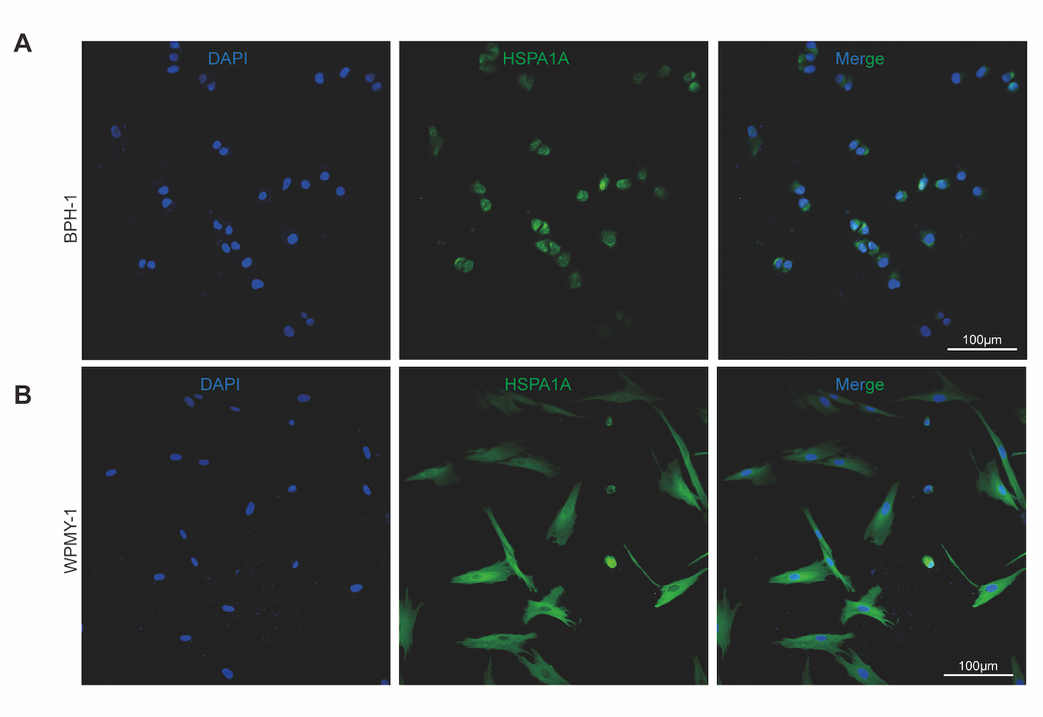


**Figure S2. Immunofluorescence staining of HSPA1A in human prostate cells.** **A** Immunoﬂuorescence staining for HSPA1A in BPH-1 cells. **B** Immunoﬂuorescence staining for HSPA1A in WPMY-1 cells. DAPI (blue) indicates nuclear staining and FITC-immunofluorescence (green) indicates HSPA1A protein staining. The scale bars are 100 μm.

**Supplementary Tables:**

**Table S1. List of primary antibodies**

| Antigens | Species antibodies  raised in | Dilution used | Supplier |
| --- | --- | --- | --- |
| HSPA1A | Rabbit, polyclonal | 1:1000 (WB)  1:500 (IF)  1:500 (IHC) | Abclonal, CHN, Cat. A0284 |
| GAPDH | Rabbit, polyclonal | 1:1000 (WB) | Abclonal, CHN, Cat. AC001 |
| CDK4 | Rabbit, monoclonal | 1:1000 (WB) | CST, USA, Cat. 12790 |
| Cyclin D1 | Rabbit, monoclonal | 1:1000 (WB) | CST, USA, Cat. 2978 |
| CDK2 | Rabbit, monoclonal | 1:1000 (WB) | Abclonal, CHN, Cat. A0094 |
| Bcl-2 | Rabbit, polyclonal | 1:1000 (WB) | Abclonal, CHN, Cat. A11025 |
| BAX | Rabbit, polyclonal | 1:1000 (WB) | Abclonal, CHN, Cat. A12009 |
| Caspase 3 | Rabbit, polyclonal | 1:1000 (WB) | Abclonal, CHN, Cat. A2156 |
| ERK1/2 | Rabbit, monoclonal | 1:1000 (WB) | Abclonal, CHN, Cat. A4782 |
| p-ERK1/2 | Rabbit, polyclonal | 1:1000 (WB) | Abclonal, CHN, Cat. AP0472 |
| JNK1/2 | Rabbit, monoclonal | 1:1000 (WB) | Abclonal, CHN, Cat. A11119 |
| p- JNK1/2  AKT  p-AKT  PI3K  p-PI3K | Rabbit, polyclonal  Rabbit, polyclonal  Rabbit, polyclonal  Rabbit, polyclonal  Rabbit, polyclonal | 1:1000 (WB)  1:1000 (WB)  1:1000 (WB)  1:1000 (WB)  1:1000 (WB) | Abclonal, CHN, Cat. AP0473  Affinity, CHN, Cat. AF3242  Affinity, CHN, Cat. AF0016  Affinity, CHN, Cat. AF6242  Affinity, CHN, Cat. AF3242 |
| NRF2 | Rabbit, monoclonal | 1:1000 (WB) | Abclonal, CHN, Cat. A21176 |
| SOD2 | Rabbit, monoclonal | 1:1000 (WB) | Abclonal, CHN, Cat. A19576 |
| CAT | Rabbit, monoclonal | 1:1000 (WB) | Abclonal, CHN, Cat. A11220 |

**Table S2. List of secondary antibodies**

| Secondary detection system used | Host | Dilution used | Supplier |
| --- | --- | --- | --- |
| Anti-Mouse-IgG (H+L)-HRP | Goat | 1:10000 (WB) | Sungene Biotech, China, Cat. #LK2003 |
| Anti-Rabbit-IgG (H+L)-HRP | Goat | 1:10000 (WB) | Sungene Biotech, China, Cat. #LK2001 |
| Anti‐rabbit IgG (H + L), F(ab')2 fragment (Alexa Fluor®488 Conjugate) | Goat | 1:50 (IF) | Cell Signaling Technology, Cat. #4412 |
| 4′,6-diamidino-2-phenylindole (DAPI) | - | 1:750 (IF) | Molecular Probes/Invitrogen, Carlsbad, CA, USA, Cat. A11007 |

**Table S3. Sense sequences of siRNAs**

| Symbol | | | (5’ to 3’) |
| --- | --- | --- | --- |
| si-con | Sense sequence | UUCUCCGAACGUGUCAG UGACAUUAAGAUUCAGGGUTT | |
| si-HSPA1A-1 | Sense sequence | GGCCUUUCCAGGUGAUCAATT  UUGAUCACCUGGAAAGGCCTT | |
| si-HSPA1A-2 | Sense sequence | GCGCAACGUGCUCAUCUUUTT  AAAGAUGAGCACGUUGCGCTT | |
| si-HSPA1A-3 | Sense sequence | CCAAGCAGACGCAGAUCUUTT  AAGAUCUGCGUCUGCUUGGTT | |

**Table S4. Primer sequences used for qRT-PCR**

| Target gene | | Human (5’ to 3’) | Tm |
| --- | --- | --- | --- |
| HSPA1A | Forward | CTGTACCAGGGTGCCGGTGGT | 62.5 |
|  | Reverse | GTCCCCAAACTCACCCTGAAGTTCT | 60.9 |
| GAPDH | Forward | GGAGCGAGATCCCTCCAAAAT | 61.6 |
|  | Reverse | GGCTGTTGTCATACTTCTCATGG | 60.9 |
